# Characterization of Biofilm-Forming Lactic Acid Bacteria from Traditional Fermented Foods and Their Probiotic Potential

**DOI:** 10.3390/foods14244299

**Published:** 2025-12-14

**Authors:** Peilin Yao, Min Kang, Mohd Esah Effarizah

**Affiliations:** 1 Food Technology Division, School of Industrial Technology, Universiti Sains Malaysia, Penang 11800, Malaysia; 2 School of Biotechnology and Food Engineering, Suzhou University, Suzhou 234000, China; 3 School of Health Management, Shanxi Technology and Business University, Taiyuan 030006, China

**Keywords:** lactic acid bacteria, biofilm, stress resistance, adhesion

## Abstract

A biofilm is a self-protective material formed by microorganisms to resist adverse environments. As an important group of microorganisms in the food industry and the human intestine, lactic acid bacteria (LAB) demonstrate enhanced probiotic activity in their biofilm state. In this study, a total of 90 LAB isolates from various traditional fermented foods across China were evaluated for their biofilm-forming capacity using the crystal violet staining method. Of these, eight isolates showed strong biofilm-forming capacity. These eight isolates were further evaluated for environmental stress responses, including tolerance to high acid and high bile salt concentrations, resistance to simulated gastrointestinal conditions, and adherence to Caco-2 cells. Four isolates with strong resistance to these stresses and adhesion to Caco-2 cells were selected for comparison between their planktonic and biofilm forms. Among these, the two isolates demonstrating the highest biofilm production capacity were AQ-4 and SY1-3, which were isolated from fermented pear juice and apple juice, respectively. Isolate AQ-4 was then identified as *Lactiplantibacillus plantarum* based on 16S rDNA sequencing. By integrating biofilm-forming capacity with key biological properties, including stress tolerance and epithelial adhesion, this study focuses on *L. plantarum* AQ-4, which exhibits distinct microstructural differences between planktonic and biofilm states, as revealed by scanning electron microscopy. The findings suggest that *L. plantarum* AQ-4 could be used to investigate the differential mechanisms in the planktonic and biofilm states and to act as the theoretical basis for the application of LAB biofilms in the food industry.

## 1. Introduction

Lactic acid bacteria (LAB) are a group of non-spore-forming, Gram-positive bacteria that ferment carbohydrates to produce lactic acid. They are widely distributed and diverse in nature [1]. As representatives of probiotics, certain LAB strains possess beneficial probiotic properties and are capable of producing functional substances such as bacteriocins, organic acids, and enzymes, including β-galactosidase and proteolytic enzymes, which could lower intestinal pH, regulate intestinal microbiota, inhibit the growth of pathogenic bacteria, prevent lactose intolerance and offer several health benefits [2,3]. LAB are also important in the production of various fermented foods such as yogurt and kimchi, which not only enhance the flavor of foods but also improve their nutritional value [4].

However, LAB typically exhibit poor resistance to environmental stresses. Their viability can be significantly compromised during food processing, transportation, and passage through the human gastrointestinal tract due to exposure to acidic, alkaline and high-temperature conditions, as well as gastric juice and bile salts [5]. Since a sufficient number of viable cells is essential for probiotic efficacy, enhancing the stress tolerance of LAB is critical for improving their survival and functional performance [6]. Currently, the common methods to enhance LAB viability include microencapsulation [7], the use of protective agents [8], supplementation with oligosaccharides [9], genetic modification [10], and biofilm formation [11].

Biofilms are structured microbial communities composed of bacterial cells embedded in a matrix of polysaccharides, proteins, nucleic acids, and lipids [12], which adhere to both abiotic or biotic surfaces. Biofilm formation is a common lifestyle for most bacteria and is widely found on medical and pathological tissues, food processing environments, and on various surfaces such as equipment and pipelines [13]. Bacteria in the biofilm state demonstrate enhanced resistance to environmental stress, making this a viable strategy for survival under adverse conditions [14]. LAB could form a biofilm, and the biofilm-forming capacity is highly strain-specific [15]. However, current studies on biofilms focus more on pathogenic bacteria and less on probiotic biofilms such as LAB. Notably, LAB biofilms have potential not only could inhibit pathogenic microorganisms but also to contribute in fermented food production [16,17].

This study aims to evaluate the biofilm-forming capacity of 90 LAB strains isolated from traditional fermented foods in China using the microtiter plate method. The outcome provides a theoretical basis for understanding LAB biofilm resistance mechanisms and for further application in industrial settings.

## 2. Materials and Methods

### 2.1. Bacterial Isolates and Culture Conditions

About 90 LAB strains were isolated from traditional fermented foods in China, including pickled vegetables, yogurts, and naturally fermented juices. Serial dilutions of the samples were prepared in sterile normal saline (0.85% NaCl) and spread onto De Man–Rogosa–Sharpe (MRS) agar plates, followed by incubation at 37 °C for 48 h. Colonies displaying typical LAB morphology (small, circular, convex, and creamy to white in appearance) were then selected and isolated using the streak plate method. Isolates exhibiting Gram-positive and catalase-negative characteristics were preliminarily screened as LAB. All isolates were preserved at −80 °C in MRS broth supplemented with 20% (*v*/*v*) glycerol until further use. Prior to experiments, the isolates were cultured in MRS broth at 37 °C for 18 h.

### 2.2. Preparation of Planktonic and Biofilm Cells

Activated strains were grown in MRS broth at 37 °C for 24 h with shaking to obtain the planktonic cells. For biofilm cells, cultures were prepared in 6-well polystyrene microtiter plates containing 4 mL MRS broth per well, to which 80 μL of bacterial suspension was added. Plates were incubated at 37 °C for 24 h. Planktonic cultures were harvested by centrifugation (12,000× *g*, 5 min), whereas biofilm cells were first gently resuspended and detached from the well surface and then collected by centrifugation under the same conditions. Collected cells were washed three times with phosphate-buffered saline (PBS), and resuspended to obtain an optical density (OD) of 0.6 at 600 nm, corresponding to ~10^8^ CFU/mL, as confirmed by plate counts [18].

### 2.3. Biofilm Formation Assay

Biofilm-forming capacity was evaluated using a stepwise workflow comprising initial OD_570_ screening, selection of representative isolates, and subsequent rescreening using the biofilm formation index (BFI). Biofilm formation was carried out based on the method described by Khan [19] with modifications. The harvested cells were adjusted to OD_600_ 0.6 and inoculated into 96-well microtiter plates in 200 µL MRS broth. After incubation at 37 °C for 24 h, the medium was removed, and each well was gently washed three times with 200 µL of sterile saline to remove planktonic cells and air dried for 30 min. Biofilms were stained with 200 µL of 0.1% crystal violet solution for 20 min, rinsed, and destained with 95% ethanol for 30 min. Absorbance at 570 nm was measured using a microplate reader, with MRS broth as the blank.

The assessment method of biofilm formation for initial screening was conducted according to Wang [20], as follows: OD_570_blank_ < OD_570_ ≤ 2OD_570_blank_ weak adherence, 2OD_570_blank_ < OD_570_ ≤ 4OD_570_blank_ moderate adherence, and OD_570_ > 4OD_570_blank_ strong adherence. The BFI was used to assess biofilm formation capacity during re-screening [21]. The BFI was calculated as follows: BFI = (A − C)/(B − D), where A is the absorbance of biofilm after staining by crystal violet at OD_570_, C is the absorbance of the blank after staining by crystal violet at OD_570_, B is the absorbance of cell concentration after incubation for 24 h at OD_600_, and D is the absorbance of blank microtiter plate at OD_600_. The biofilm-forming capacity of the isolates was classified as follows: BFI < 0.7 weak adherence, 0.7 ≤ BFI ≤ 1.6 moderate adherence, and BFI ≥ 1.7 strong adherence.

### 2.4. Observation Using the Fluorescent Inverted Microscope

A coverslip was placed in a 6-well plate containing MRS broth and bacterial suspension. After inoculation at 37 °C for 24 h, the coverslip was gently washed with sterile PBS to remove unattached cells and stained with 20 µM carboxyfluorescein diacetate succinimidyl ester (CFDA-SE) and 0.5 µM of propidium iodide (PI) for 30 min. The coverslip was gently washed with PBS to remove excess dye [22]. The structure of biofilm formation was observed using inverted fluorescent microscope (IX71, OLYMPUS, Tokyo, Japan).

### 2.5. Acid Tolerance Assay

Acid tolerance was assessed following Oh & Jung [23] with some modifications. MRS broth was adjusted to pH 2.0–7.0 using 0.1 mol/L HCl, and 200 µL of bacterial suspension was added into 10 mL broth. The samples were incubated at 37 °C for 24 h. The turbidity of bacterial solution was determined using the microplate reader at 600 nm. The group with pH value of 7.0 was taken as the control group. Experiments were performed in triplicate.

### 2.6. Bile Salt Tolerance Assay

Bile salt tolerance was determined in MRS broth supplemented with 0.1–0.4% bile salts, with unsupplemented broth as the control [23]. Bacterial suspensions (200 µL) were inoculated into 10 mL of broth and incubated at 37 °C for 24 h. Absorbance was measured at 600 nm, and survival of bacteria in bile salt was expressed as A/A_0_ × 100%, where A_0_ and A represent the absorbance of the control and experimental groups, respectively.

### 2.7. In Vitro Gastrointestinal Tolerance Assay

In vitro gastrointestinal tolerance assays were performed as described by Minkus [24] with some modifications. Simulated gastric fluid (SGF) and simulated intestinal fluid (SIF) were freshly prepared before the experiment. Pepsin and trypsin were used as supplied by the manufacturer, and enzyme activities were not independently verified. For SGF, hydrochloric acid was diluted with sterile distilled water to obtain a solution with pH 2.0. Then, pepsin was added to the solution at a final concentration of 10 g/L. For SIF, 6.8 g KH_2_PO_4_ was dissolved in 500 mL of sterile distilled water. The pH was adjusted to 6.8 by NaOH solution. Sterile distilled water was added to the mixture to the volume of 1000 mL. After enzyme addition, the pH of both SGF and SIF was re-checked using a calibrated pH meter and readjusted when necessary. Finally, 3 g of bovine bile salt and 10 g of trypsin were mixed into the solution. All SGF and SIF preparations were filtered using the microporous filter membrane (pore size 0.22 µm) before the tolerance assays.

The bacterial suspension was adjusted to OD_600_ 1.0, and then 1 mL of the bacterial suspension was added to 9 mL of SGF or SIF. The samples were incubated in a water bath at 37 °C for 2 h with gentle agitation (approximately 100 rpm) every 30 min. The viable bacterial count from the solutions was determined by the conventional plate counting method on an MRS agar plate. All experiments were performed in triplicate. Survival of the bacteria in SGF/SIF was expressed in terms of B/B_0_ × 100%, where B_0_ and B represent the viable log (CFU/mL) samples before and after digestion.

### 2.8. Adhesion to Caco-2 Cells Assay

Caco-2 cells at passage 5 (BeNa Culture Collection, Shanghai, China) were grown to 80%, trypsinized and added to a 6-well microtiter plate with coverslips [25]. Then, 2 mL of complete cell culture suspension was added to each well. The samples were incubated in a 5% CO_2_ incubator at 37 °C until the cells were attached to the surface of the wells. After removing the medium, 1 mL of bacterial suspension and 2 mL of basal cell culture solution were added to each well and incubated at 37 °C for 2 h. The bacterial suspensions were added at a multiplicity of infection (MOI) of 100:1 (bacteria/cell). After incubation, non-adherent bacteria were removed by washing with PBS, and adhesion was quantified based on bacteria attached to the cell monolayer only. The samples were fixed in methanol for 20 min and stained with Gram-staining. Twenty random microscopic fields were examined, and the number of bacteria adhered per 100 Caco-2 cells was recorded as the adhesion index.

### 2.9. Identification of Bacteria

16S rDNA sequencing analysis was used to identify the strains. Genomic DNA was extracted using rapid bacterial genomic DNA isolation kit (Sangon Biotech, Shanghai, China). The 16S rDNA was amplified with the universal bacterial primers 27F/1492R under the following conditions: 32 cycles of 94 °C for 5 min, 94 °C for 1 min, 53 °C for 30 s, 72 °C for 1 min, and final extension at 72 °C for 5 min. PCR products were analyzed by electrophoresis on 1% agarose gel (120 V, 40 mA, 30 min) with a 200–2000 bp maker. The successful amplified fragment was sequenced by Sangon Biotech (Shanghai, China), and sequences were analyzed using National Center for Biotechnology Information (NCBI) BLAST Version 2.2.26. Phylogenetic trees were generated using MEGA Version 12.1 software.

### 2.10. Scanning Electron Microscopy (SEM) of Planktonic and Biofilm Bacteria

Planktonic and biofilm cells (prepared as in Section 2.2) were fixed in 2.5% glutaraldehyde for 12 h, dehydrated in graded ethanol (30%, 50%, 70%, 90%, and 100%), and freeze-dried. Samples were sputter-coated a layer of gold and observed under SEM (2100F, JEOL, Tokyo, Japan).

### 2.11. Statistical Analysis

Statistical significance of results was calculated using one-way ANOVA in SPSS 23.0 software, followed by Duncan’s test for the least significant difference at a significance level of *p* < 0.05. The bars represent data from three independent experiments. Values are presented as the mean ± SD (n = 3).

## 3. Results

### 3.1. Ability of LAB to Form Biofilms

A microtiter plate assay was conducted to investigate the biofilm formation capacity of 90 LAB strains isolated from traditional fermented foods in China. The blank OD_570_ was 0.136 in the initial screening experiment, and the classification thresholds (weak: 0.136–0.271; moderate: 0.272–0.544; strong: >0.544) reflected multiples of the blank to normalize baseline staining. Using this approach, 15 isolates showed strong biofilm-forming capacity (18.89%), 13 isolates with moderate biofilm-forming capacity (13.33%), and 62 isolates with weak biofilm-forming capacity (67.78%) after 24 h incubation, as shown in Table 1. These results indicate that most LAB isolates were relatively weak in biofilm production, although a discrete subset showed markedly stronger adherence. To confirm the reliability of these findings, all 15 isolates classified as strong biofilm formers were rescreened using the BFI, which incorporates both adherent biomass (OD_570_) and growth (OD_600_). In addition, five isolates each from the moderate and weak biofilm-forming groups were randomly included solely for comparative validation and to minimize misclassification arising from growth-rate differences. This rescreening identified 8 isolates with consistently strong biofilm-forming capacity (1-1, SN-8, SY2-3, SY1-3, SN-6, G-2, AQ-4, and 20250) as shown in Table 2. Among them, SY1-3 exhibited the highest BFI, followed by AQ-4, SY2-3, and 1-1.

### 3.2. Fluorescent Microscope Assay

**Figure 1:**
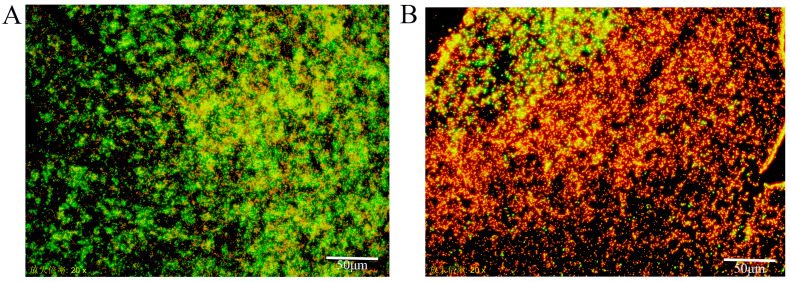
Biofilm structure of LAB strains observed with an inverted fluorescence microscope (×200 magnification). (**A**) Strain AQ-4 formed a dense, mushroom-like biofilm composed mainly of viable cells (green fluorescence, CFDA-SE). (**B**) Strain 20250 developed an intact biofilm with predominantly non-viable cells (red fluorescence, propidium iodide).

The biofilm structures of 25 isolates representing different formation capacities were observed under a fluorescent microscope. Only the AQ-4 and 20250 strains formed typical biofilm structures on glass slides, with the bacterial cells connected tightly in a clumped mushroom shape and displaying an intact biofilm structure (Figure 1). Live/dead staining showed that AQ-4 biofilms consisted largely of viable cells (green), while 20250 biofilms contained predominantly dead cells (orange). The remaining isolates did not form typical biofilm structures on the glass slides, and the bacterial cells were distributed in a scattered pattern. AQ-4’s formation of a dense, viable biofilm highlights its superior ability to persist under sessile conditions.

### 3.3. Tolerance of LAB with High Biofilm Production to Different Types of Environmental Stress

LAB strains with good probiotic properties could resist different types of environmental stress. This study measured the survival rates of the strains under acid, bile salt, and artificial gastric fluid treatments. The results are shown in Table 3, Table 4 and Table 5.

At pH < 4, growth was significantly reduced for all strains (*p* < 0.05) whereas at pH ≥ 4 growth was similar among strains. At pH 2, strains SN-6 and 20250 exhibited the strongest acid tolerance, followed by strains G-2, SN-8, and AQ-4. Meanwhile, at pH 3, the SY1-3 strain exhibits the strongest acid tolerance, followed by strains SN-6, AQ-4, and 20250 (Table 3).

The results of bile salt tolerance (Table 4) showed that the survival rates of the strains decreased as the bile salt concentrations increase (1–4 g/L). Except for the bile salt concentration of 1 g/L, the survival rates of the strains at other concentrations differed greatly. The strains SN-8, AQ-4, and G-2 display the highest survival rates at the concentration of 1 g/L. AQ-4 has the strongest tolerance rate at the concentration of 2 g/L, followed by SN-8, SY1-3, and G-2. Likewise, AQ-4 exhibits the strongest tolerance rate, followed by SN-8 and G-2 at a concentration of 3 g/L. Meanwhile, SN-8 reveals the strongest tolerance rate, followed by SN-8 and G-2 at the concentration of 4 g/L. Since these bile levels correspond to physiological small intestinal conditions, these findings suggest that strains such as AQ-4 and SN-8 are more like to survive the bile challenge in vivo.

After 2 h in simulated gastric fluid (SGF), survival was highest for 20250 (29.3%) and SN-8 (28.7%), followed by SY2-3 (23.75%) (Table 5). Survival was consistently higher in simulated intestinal fluid (SIF) than in SGF. AQ-4 exhibited the strongest survival in SIF (75.95%), followed by SY1-3 (65.2%) and SN-8 (62.2%). These results confirm that SGF is the major barrier for LAB survival, while the intestinal environment is comparatively less hostile. Notably, AQ-4 combines moderate gastric survival with superior intestinal persistence, suggesting niche-specific adaptation.

### 3.4. Adherence to Caco-2 Cells

**Figure 2:**
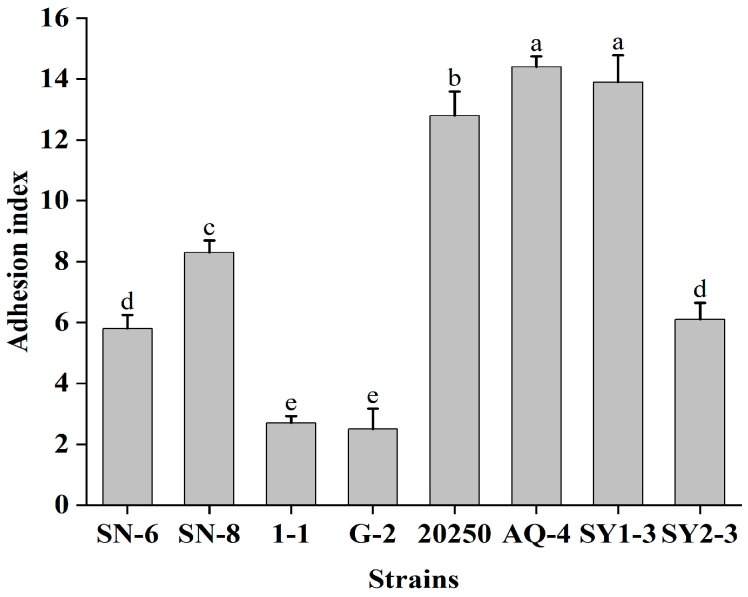
Adhesion of eight LAB strains with high biofilm-forming capacity to Caco-2 cells. The adhesion index was calculated as the number of bacterial cells adhering per 100 Caco-2 cells. Significant differences (*p* value < 0.05) are indicated by different lowercase letters.

In this study, eight LAB strains with high biofilm production were investigated for their ability to adhere to Caco-2 cells, as shown in Figure 2. A significant difference in the adhesion to Caco-2 cells between the strains was observed, with AQ-4, SY1-3, and 20250 showing excellent adhesion (index of ≥12), while G-2 and 1-1 showing poor adhesion. The strains with excellent adhesion in this study may correlate with the important trait for colonization and pathogen exclusion.

### 3.5. Stress Tolerance in Planktonic vs. Biofilm States

**Figure 3:**
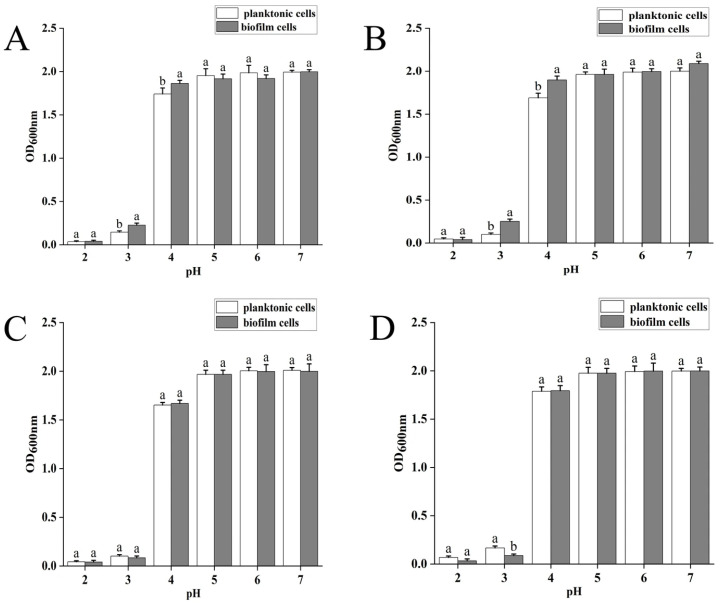
Acid tolerance of four LAB strains in planktonic and biofilm states at different pH levels. (**A**) strain SY1-3; (**B**) strain AQ-4; (**C**) strain 20250; (**D**) strain SN-8. Significant differences (*p* value < 0.05) are indicated by different lowercase letters.

Acid tolerance of the four LAB strains in the planktonic and biofilm states is shown in Figure 3. The extremely acidic environment inhibits the growth of the strains at a pH of 2.0–3.0. However, AQ-4 and SY1-3 show significantly higher growth among the biofilm strains than the planktonic strains at pH 3.0 and 4.0. Meanwhile, SN-8 grew better in the planktonic state than the biofilm state at pH 3.0, probably due to the denaturation of associated acid-sensitive stress proteins and loss of activity under an extremely acidic environment, leading to structural changes in the biofilm and antagonism between the cells. These results indicate that the biofilm form has a protective effect on SY1-3 and AQ-4 at pH 3.0 and 4.0.

**Figure 4:**
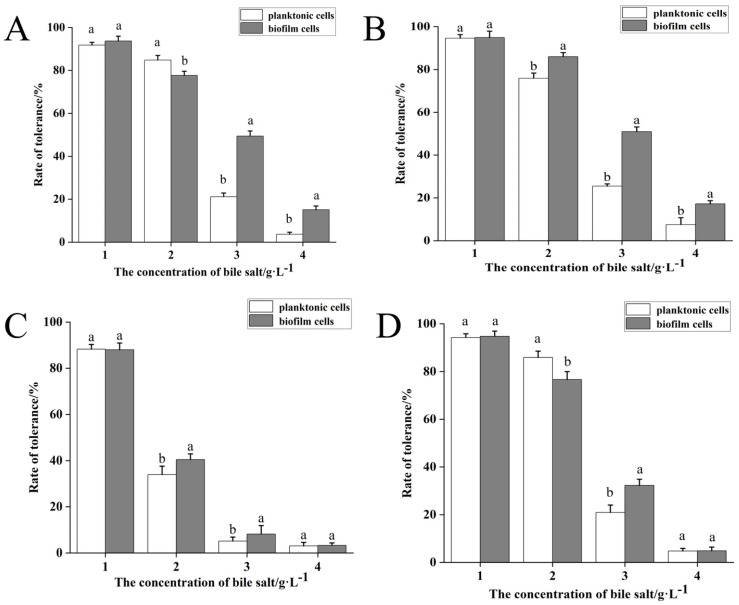
Survival of four LAB strains in planktonic and biofilm states at increasing bile salts concentrations. (**A**) strain SY1-3; (**B**) strain AQ-4; (**C**) strain 20250; (**D**) strain SN-8. Significant differences (*p* value < 0.05) are indicated by different lowercase letters.

The rate of bile salt tolerance of four LAB strains in the planktonic and biofilm states is shown in Figure 4. The tolerance rate of the strains decreased as the bile salt concentration increases at 1–4 g/L concentrations. No significant difference was observed between the planktonic and biofilm forms at the bile salt concentration of 1 g/L. In contrast, the tolerance rates are significantly higher in the planktonic form than in the biofilm form for AQ-4 and 20250 at the bile salt concentration of 2 g/L. The biofilm form has significantly higher tolerance rates than the planktonic form for the four LAB strains at the bile salt concentration of 3 g/L, indicating that biofilm formation could improve bile salt tolerance. However, at the bile salt concentration of 4 g/L, only SY1-3 and AQ-4 show significantly higher growth in the biofilm state than in the planktonic state, indicating that the biofilm form played a protective role for the strains. Although biofilm-associated cells generally showed increased tolerance to environmental stresses, this effect was strain dependent rather than universal. For example, strain SN-8 did not consistently show improved tolerance in the biofilm state under acidic or bile salt conditions, indicating that biofilm-mediated protection varies among LAB strains.

**Figure 5:**
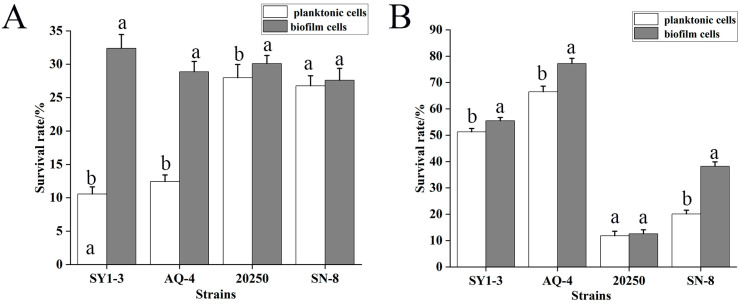
Survival of LAB strains in planktonic and biofilm states after 2 h in stimulated gastrointestinal fluid. Biofilm cells showed improved tolerance compared with planktonic cells, except SN-8. (**A**) gastric fluid; (**B**) intestinal fluid. Significant differences (*p* value < 0.05) are indicated by different lowercase letters.

The survival rates after 2 h of culture in gastric fluid and intestinal fluid are shown in Figure 5A,B, respectively. For gastric fluid, except for SN-8, the biofilm form has a significantly higher survival rate than the planktonic form, with the survival rates of the former being 3.07, 2.32, and 1.08 times higher than in the latter form for strains SY1-3, AQ-4, and 20250. These results indicate that biofilm formation effectively improved the tolerance of the strains to gastric juice stress. Biofilm SY1-3 has the highest survival rate in gastric fluid, reaching 32.4%. For intestinal fluid, all three strains are significantly more viable in the biofilm form than in the planktonic form, except for the strain 20250. The survival rate of strain AQ-4 in the intestinal juice is higher than other strains in both forms, reaching 66.50% and 77.21%. Therefore, biofilm formation improved the tolerance of the strains to intestinal fluid stress.

### 3.6. Adherence to Caco-2 Cells in the Planktonic and Biofilm States

**Figure 6:**
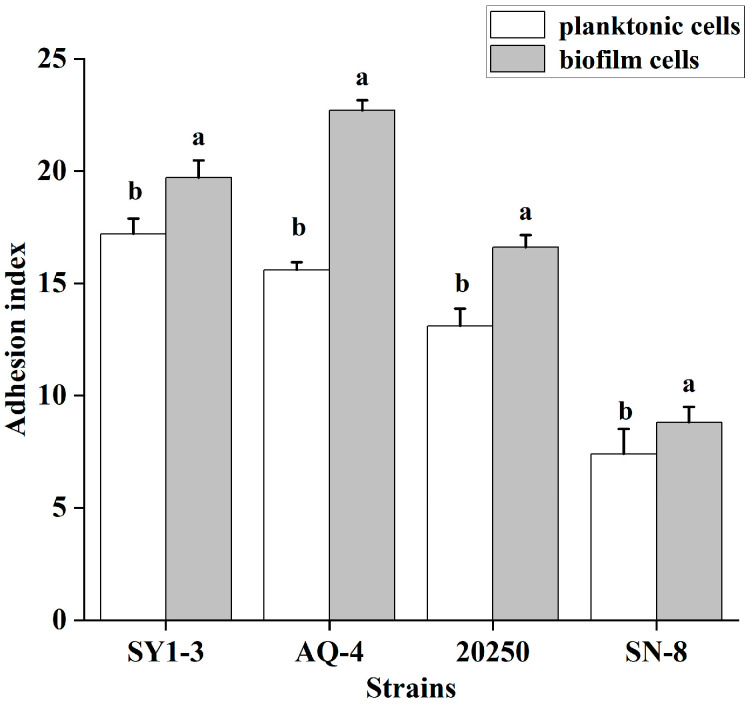
Adhesion indices of four LAB strains in planktonic and biofilm strains to Caco-2 cells. The adhesion index refers to the number of bacteria adhered per 100 Caco-2 cells. Significant differences (*p* value < 0.05) are indicated by different lowercase letters.

The adhesion index of both forms to Caco-2 cells was measured to investigate the ability of the strains to adhere to the intestine in different forms. The results are shown in Figure 6. The adhesion index of the biofilm form to Caco-2 cells increases compared to the planktonic form for the four strains, with AQ-4 showing the most significant improvement. Therefore, the biofilm form improved the ability of the strains to adhere to the intestine, providing the necessary conditions for the growth and colonization of LAB in the intestine.

### 3.7. Identification of LAB

**Figure 7:**
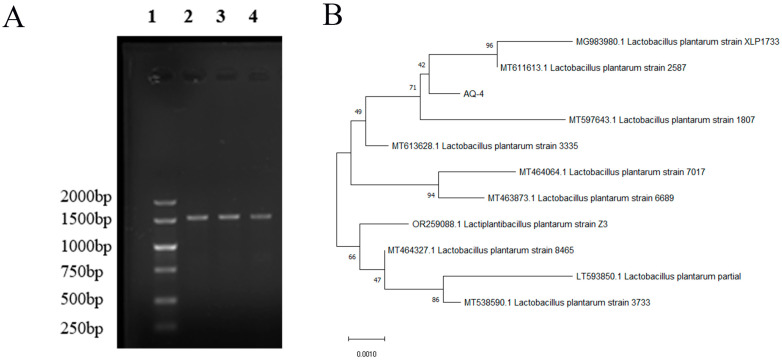
Molecular identification of *Lactiplantibacillus plantarum* (previously known as *Lactobacillus plantarum*) AQ-4. (**A**) Agarose gel electrophoresis of amplified 16S rDNA. (**B**) Phylogenetic tree based on 16S rDNA sequences, constructed using MEGA software Version 12.1. Scale bar 0.01 indicates the nucleotide substitution rate at each site. Bootstrap probabilities were presented as the percentage values and determined using 1000 replicates. Before the strain name, the existing numbers represent the accession numbers of selected sequences.

Based on the results of biofilm-forming capacity and biological characteristics, one strain (AQ-4) with excellent performance was selected. In this study, AQ-4 was the only strain selected for molecular identification by the 16S rDNA sequencing. A phylogenetic tree was generated based on its 16S rDNA sequence (Figure 7). The results show that the isolated LAB strain belonged to *Lactiplantibacillus plantarum*, with 99% similarity.

### 3.8. Microstructure of L. plantarum AQ-4 in Different States

**Figure 8:**
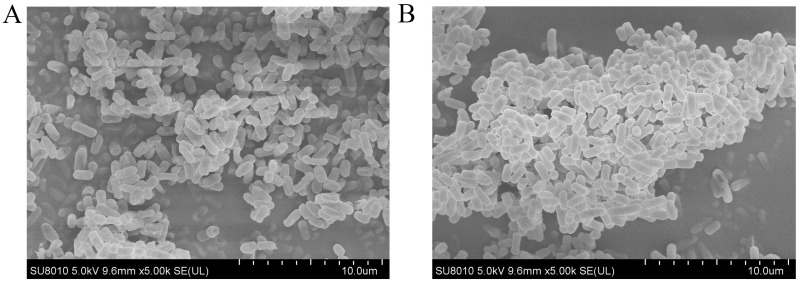
SEM images of *Lactiplantibacillus plantarum* AQ-4 in different states under ×5000 magnification. (**A**) Planktonic cells appear scattered and loosely associated. (**B**) Biofilm cells form dense, three-dimensional clusters with typical biofilm structure.

The microstructure of *L. plantarum* AQ-4 in the planktonic and biofilm states was observed by SEM, as shown in Figure 8. The clumped structure formed by the planktonic strain is small, with loose wrapping between the bacteria, distributed in a fragmented state. On the contrary, the clusters formed are large and tight in the biofilm state, forming a typical three-dimensional biofilm structure. It indicates a varied microstructure of AQ-4 in the biofilm and planktonic states.

## 4. Discussion

A microtiter plate format assay was used to investigate the biofilm formation capacity of 90 LAB strains isolated from Chinese traditional fermented foods. While most isolates were weak biofilm formers, a subset showed strong adherence, with OD_570_ values up to 2.402. Based on initial screening, twenty-five strains (15 strong, 5 moderate, 5 weak) were selected for rescreening using the biofilm formation index (BFI), resulting in eight LAB strains being confirmed as strong biofilm producers. Fluorescent microscopy further revealed that only AQ-4 and 20250 formed typical mushroom-like biofilm structures on glass slides, whereas other isolates appeared as scattered cells. This suggests that only certain strains combine high biomass with stable architecture, and that surface type strongly influences biofilm morphology.

Beyond biofilm formation itself, probiotic functionality requires survival under gastrointestinal stress conditions. LAB are generally sensitive to adverse conditions such as low pH, heat, bile salts, and digestive enzymes, all of which reduce cell viability during processing, storage and gastrointestinal transit. Because a sufficient viable population is a prerequisite for probiotic efficacy [26], only strains with both strong biofilm capacity and stress resistance are likely to be effective in vivo. Toscano et al. [27] proposed ten golden rules for the proper use of probiotics, including selecting probiotic strains that are resistant to the gastrointestinal environment, and the probiotic strains must be able to colonize the gut.

Accordingly, the present study evaluated stress tolerance as a functional extension of biofilm screening. Low pH poses major challenges for LAB, as intracellular acidification inactivates enzymes, disrupts metabolism, and damages macromolecules [28]. On the other hand, bile salts compromise membrane integrity and permeability [29]. Gastric juice represents the most formidable barrier, with highly acidic conditions (pH as low as 1.5 in fasting states) and the presence of pepsin [30,31]. Most microorganisms are rapidly killed under these conditions, and only acid-tolerant LAB can reach the intestine [32]. The intestinal fluid is slightly alkaline, with main components of trypsin and bile salts, and the mass fraction of bile salt is generally maintained at 0.03% to 0.30%, which has a coercive effect on the growth of LAB [33]. For probiotic function, viable LAB populations of at least 10^8^ CFU/mL are generally required [34]. Adhesion to epithelial surfaces is equally important, as it promotes colonization and competitive exclusion of pathogens [35]. Caco-2 and HT-29 cells are widely used to screen this ability in vitro [36]. In this study, eight LAB strains with strong biofilm formation were further tested for acid, bile, and gastrointestinal tolerance as well as adhesion to Caco-2 cells. Four strains demonstrated consistently high resistance and adhesion, making them promising candidate for probiotic use.

The enhanced performance of these strains can be attributed to the protective role of biofilm formation. Biofilm formation provides a natural protective mechanism, with extracellular polymers creating diffusion barriers and buffering cells from environmental stress. Previous studies have shown that biofilm cells differ from planktonic cells in morphology and physiology, often exhibiting greater resistance to ethanol and organic acids [37,38,39]. Our results align with these observations, as LAB biofilm cells displayed improved tolerance to acid, bile and simulated digestive fluids compared to planktonic forms. The enhanced adhesion observed for selected biofilm-associated LAB strains may be attributed to several strain-dependent mechanisms. During biofilm formation, the production of extracellular polymeric substances (EPS) can promote cell–surface interactions and stabilize attachment to epithelial cells. In addition, surface-associated proteins, including adhesins and mucus-binding proteins, may be upregulated in biofilm-associated cells, thereby facilitating stronger interactions with host epithelial surfaces. Quorum sensing systems have also been reported to regulate both biofilm formation and adhesion-related gene expression, suggesting coordinated control of colonization-related traits in certain LAB strains. Disruption of quorum sensing genes such as *luxS* has been shown to reduce adhesion in *L. plantarum* [40]. These findings underscore that biofilm competence is not only strain-specific but also directly tied to probiotic functionality.

From the current study, two strains with strong environmental stress tolerance and adhesion ability in the biofilm state were screened, and AQ-4 was selected as the most promising candidate. Identified by 16S rDNA as *L. plantarum*, this strain displayed robust biofilm formation, high survival under gastrointestinal stress, and strong epithelial adhesion. SEM confirmed distinct morphological differences between planktonic and biofilm states whereby the planktonic cells were dispersed, while biofilm cells formed dense, three-dimensional clusters. These structural features provide a clear explanation for the functional advantages observed.

The discrepancy observed between strong biofilm formation in the microtiter plate assay and weak or incomplete structural assembly on glass slides for some strains may be attributed to strain-specific differences in surface properties and adhesion behavior. Biofilm formation assessed by crystal violet staining primarily reflects total adherent biomass on polystyrene surfaces, whereas biofilm development on glass is more strongly influenced by physicochemical interactions between the bacterial cell surface and the abiotic substrate [41,42]. Variations in cell surface hydrophobicity, charge distribution, and extracellular polymeric substance (EPS) composition among LAB strains may therefore result in distinct biofilm architectures on different surfaces, explaining why only certain strains formed well-organized structures on glass slides.

Strain-dependent tolerance to environmental stress may also be closely related to metabolic activity and biofilm microstructure. Dense, three-dimensional biofilm architectures can restrict the penetration of adverse environmental factors and create localized microenvironments that support metabolic stability and stress adaptation. In contrast, loosely structured or fragmented biofilms may provide limited protection, resulting in variable tolerance even among strains exhibiting high biofilm biomass in microtiter assays [14,38].

The SEM-observed biofilm architecture of *Lactobacillus plantarum* AQ-4, characterized by compact cell aggregates embedded within an extracellular matrix, is consistent with previously reported *L. plantarum* biofilm morphotypes [37,43]. Such morphologies have been associated with enhanced resistance to acidic and bile stress, supporting the superior tolerance of AQ-4 in the biofilm state observed in this study.

This study provides novel insights into LAB biofilm biology by focusing on *L. plantarum* AQ-4 as a model strain with pronounced biofilm-forming capacity. Unlike many studies that primarily report biofilm formation based on quantitative assays, this work integrates structural characterization using SEM with functional biofilm-related assays. Furthermore, by directly comparing planktonic and biofilm states, this study highlights distinct physiological and structural adaptations associated with biofilm formation. Collectively, these findings contribute to a more comprehensive understanding of LAB biofilm behavior and support the potential application of biofilm-forming *L. plantarum* strains in food-related and industrial contexts.

## 5. Conclusions

Of the 90 LAB isolates tested, eight strains displayed strong biofilm formation, with AQ-4 (*L. plantarum*) emerging as the most promising probiotic candidate. It has strong resistance to acid, bile salts, and artificial gastrointestinal fluids, as well as strong adhesive ability to Caco-2 cells. This research provides excellent strain resources for exploring the differences between planktonic and biofilm cell characteristics and a theoretical basis for the development and application of LAB biofilms while simultaneously serving as a guide for the development of high-efficiency probiotic micro-preparations.

## Figures and Tables

**Table 1:** Distribution of biofilm formation capacity of 90 LAB isolates from traditional fermented foods.

Isolation Source (n)	Specific Source	Strong (OD_570_ > 0.544)	Moderate (OD_570_ 0.272–0.544)	Weak (OD_570_ 0.136–0.271)
Pickled vegetables (62)	Cabbage, radish, cucumber, asparagus lettuce, mustard leaf, carrot, yam, pepper, celery, green bean	H-2, 20250, 1-1, 1-2, 7-1, A-1, A-2, B-1, G-2	10-4, PCB-3, 14-2, 10-3, 2-1, 4-3, B-3, G-3, I-3, PCB-1, PCB-4	2-2, 3-1, 3-2, 3-3, 3-5, 4-1, 4-2, 4-4, 5-1, 5-2, 5-3, 5-4, 6-1, 6-2, 6-3, 6-4, 7-2, 7-3, 7-4, 9-1, 9-2, 9-3, 10-1, D-1, D-2, F-3, J-1, Q-1, Q-2, Q-3, Q-4, Q-5, FG-1, NO, PT1, PT2, PCB-2, PCH-1, PCH-2, PCG-1, PCG-2, PCG-3
Yogurt (11)	Fermented milk products originated from Tibet regions of China.	SN-8, SN-6, YLD-LC	–	XN-3, XN-4, D1, SN-1, SN-2, XN-1, XN-2, XN-5
Fermented juices (17)	Pear, apple, grape, pomegranate, fig	SY2-3, AQ-4, SY1-3	AQ-5, AQ-7	AQ-2, AQ-3, AQ-6, 21805, SC-2, SC-3, LMJS-1, SY1-1, SY2-1, SY2-2, SY3-1, SY1-4
Total (90)		15 (16.7%)	13 (14.4%)	62 (68.9%)

Isolate IDs listed in each table entry represent isolates classified as strong, moderate, or weak biofilm formers, grouped by isolation source. Classification thresholds: weak (0.136 < OD_570_ ≤ 0.272); moderate (0.272 < OD_570_ ≤ 0.544); strong (OD_570_ > 0.544).

**Table 2:** Re-screening results of biofilm formation capacity of 25 LAB isolates.

Product Category	Specific Source	Isolate ID	BFI (Mean ± SD)	Biofilm Formation Capacity
pickled vegetable	cabbage	1-1	5.512 ± 0.102	strong
	cabbage	1-2	0.073 ± 0.012	weak
	cabbage	A-1	0.442 ± 0.034	weak
	cabbage	A-2	0.081 ± 0.009	weak
	radish	2-1	0.570 ± 0.037	weak
	cucumber	4-3	0.523 ± 0.045	weak
	pepper	G-2	3.094 ± 0.072	strong
	carrot	20250	2.980 ± 0.055	strong
	yam	PCG-1	1.324 ± 0.036	moderate
	yam	PCB-3	1.509 ± 0.057	moderate
	mustard leaf	10-4	0.880 ± 0.019	moderate
	mustard leaf	10-3	0.357 ± 0.024	weak
	celery	H-2	0.378 ± 0.035	weak
	radish	B-1	0.169 ± 0.013	weak
	asparagus lettuce	7-1	0.436 ± 0.037	weak
	green bean	PT1	0.567 ± 0.023	weak
naturally fermented juice	pear	AQ-4	6.181 ± 0.077	strong
	apple	SY2-3	5.645 ± 0.087	strong
	apple	SY1-3	10.206 ± 0.115	strong
	fig	21805	0.760 ± 0.039	moderate
yogurt	milk	SN-8	4.924 ± 0.098	strong
	milk	SN-6	4.339 ± 0.091	strong
	milk	XN-1	0.114 ± 0.021	weak
	milk	YLD-LC	0.106 ± 0.015	weak
	milk	D1	0.102 ± 0.008	weak

Isolate IDs are listed with their biofilm formation index (BFI), classified as strong (BFI ≥ 1.7), moderate (0.7–1.6), or weak (BFI < 0.7).

**Table 3:** Effect of pH on the growth of eight LAB strains with strong biofilm-forming capacity.

	pH2	pH3	pH4	pH5	pH6	pH7
1-1	0.111 ± 0.004 ^a^	0.107 ± 0.002 ^a^	1.873 ± 0.14	1.949 ± 0.07	1.959 ± 0.11	1.976 ± 0.02
G-2	0.154 ± 0.005 ^b^	0.304 ± 0.007 ^b^	1.922 ± 0.11	1.961 ± 0.04	1.971 ± 0.08	1.977 ± 0.06
SN-6	0.183 ± 0.003 ^c^	0.466 ± 0.02 ^c^	1.833 ± 0.07	1.949 ± 0.09	1.959 ± 0.07	1.976 ± 0.08
SN-8	0.153 ± 0.006 ^b^	0.299 ± 0.008 ^b^	1.855 ± 0.07	1.959 ± 0.12	1.97 ± 0.03	1.978 ± 0.06
SY1-3	0.129 ± 0.002 ^d^	0.52 ± 0.04 ^d^	1.841 ± 0.09	1.936 ± 0.05	1.961 ± 0.13	1.966 ± 0.03
SY2-3	0.123 ± 0.005 ^d^	0.375 ± 0.03 ^e^	1.833 ± 0.08	1.965 ± 0.08	1.97 ± 0.06	1.976 ± 0.10
AQ-4	0.154 ± 0.003 ^b^	0.461 ± 0.04 ^c^	1.875 ± 0.12	1.932 ± 0.06	1.952 ± 0.08	1.958 ± 0.07
20250	0.181 ± 0.004 ^c^	0.432 ± 0.02 ^c^	1.807 ± 0.06	1.952 ± 0.12	1.966 ± 0.09	1.981 ± 0.08

Values represent mean OD_600_ after 24 h of triplicate experiments. Different lowercase letters indicate significant differences between different strains at the same pH value (*p* < 0.05).

**Table 4:** Survival of eight LAB strains in MRS broth supplemented with different concentrations of bile salt (0.1–0.4%).

	0.1%	0.2%	0.3%	0.4%
1-1	86.90 ± 1.56 ^d^	12.57 ± 0.56 ^d^	10.00 ± 0.45 ^d^	8.33 ± 0.35 ^d^
G-2	96.22 ± 1.34 ^a^	79.21 ± 1.23 ^b^	56.64 ± 1.02 ^b^	25.26 ± 0.46 ^c^
SN-6	94.57 ± 1.89 ^b^	6.40 ± 0.58 ^e^	6.30 ± 0.77 ^e^	5.23 ± 0.29 ^e^
SN-8	97.54 ± 1.23 ^a^	81.26 ± 1.32 ^b^	57.85 ± 1.18 ^b^	55.15 ± 0.77 ^a^
SY1-3	94.15 ± 1.45 ^b^	79.36 ± 1.17 ^b^	42.58 ± 1.21 ^c^	43.83 ± 0.62 ^b^
SY2-3	93.20 ± 1.67 ^b^	6.53 ± 0.77 ^e^	6.20 ± 0.55 ^e^	5.40 ± 0.33 ^e^
AQ-4	96.79 ± 1.22 ^a^	84.41 ± 1.76 ^a^	65.95 ± 0.89 ^a^	44.46 ± 0.49 ^b^
20250	90.67 ± 1.08 ^c^	58.71 ± 1.12 ^c^	43.20 ± 0.75 ^c^	24.96 ± 0.32 ^c^

Values are expressed as survival percentage (A/A_0_ × 100%). Different lowercase letters indicate significant differences between different strains at the same concentration of bile salt (*p* < 0.05).

**Table 5:** Survival of eight LAB strains after 2 h incubation in simulated artificial gastric fluid and intestinal fluid (SGF, pH 2.0 with pepsin) and simulated intestinal fluid (SIF, pH 6.6 with bile salts and trypsin).

	1-1	G-2	SN-6	SN-8	SY1-3	SY2-3	AQ-4	20250
Gastric fluid	1.40 ± 0.02 ^e^	4.15 ± 0.06 ^d^	4.00 ± 0.05 ^d^	28.70 ± 0.36 ^a^	10.50 ± 0.32 ^c^	23.65 ± 0.38 ^b^	11.70 ± 0.19 ^c^	29.30 ± 0.28 ^a^
Intestinal fluid	48.8 ± 0.22 ^c^	46.2 ± 0.45 ^c^	15.5 ± 0.12 ^e^	62.2 ± 0.55 ^b^	65.2 ± 0.42 ^b^	30.3 ± 0.27 ^d^	75.95 ± 0.62 ^a^	47.7 ± 0.45 ^c^

Values are expressed as survival percentage (B/B_0_ × 100%). Different lowercase letters indicate significant differences between different strains in the gastric fluid or intestinal fluid (*p* < 0.05).

## Data Availability

The original contributions presented in this study are included in the article. Further inquiries can be directed to the corresponding author.
